# Intracranial hypertension after MECT: successful transition to rTMS for refractory auditory hallucinations: a case report

**DOI:** 10.3389/fpsyt.2026.1841894

**Published:** 2026-07-13

**Authors:** Liang Lv, Meng-Yun Guan

**Affiliations:** Department of Psychiatry, Huzhou Third Municipal Hospital, Huzhou, Zhejiang, China

**Keywords:** adverse events, antipsychotic medications, intracranial hypertension syndrome, modified electroconvulsive therapy, refractory auditory hallucinations, repetitive transcranial magnetic stimulation

## Abstract

A 21-year-old female patient with schizophrenia, whose primary symptom was refractory auditory hallucinations, developed intracranial hypertension syndrome following her first session of modified electroconvulsive therapy (MECT), presenting with severe headache, projectile vomiting, diplopia and meningeal signs. She subsequently received 1 Hz repetitive transcranial magnetic stimulation (rTMS) applied to the left temporo-parietal cortex with each session delivering 1,500 pulses at 74% of the resting motor threshold. Her auditory hallucinations diminished markedly 3 days after completing rTMS and resolved completely 1 week later, accompanied by improved social functioning. No recurrence was observed during 3 weeks of follow-up. This case primarily demonstrates that rTMS may represent a safe and effective alternative for patients with refractory auditory hallucinations who are intolerant to MECT. It serves as a clinical reminder to maintain vigilance for elevated intracranial pressure following MECT, particularly in patients with a history of migraine.

## 1 Introduction

Schizophrenia is a severe chronic mental disorder characterized primarily by psychotic symptoms such as hallucinations and delusions, as well as cognitive and emotional impairments (1). Auditory hallucinations (AH) are the most common and central symptom of the disorder, with approximately 60%–80% of patients experiencing them at some point during their illness (2). Persistent AH severely impairs patients’ daily functioning and social interactions, significantly reduce their quality of life (3), and are closely associated with poor medication adherence (4) and an increased risk of suicide (5).

Antipsychotic medications are the first-line treatment for AH; approximately 25–50% of patients experience persistent hallucinations despite good medication adherence (6). While clozapine is highly effective in reducing AH and suitable for long-term use, over 25% of patients remain symptomatic (7). For treatment-resistant cases, modified electroconvulsive therapy (MECT) is effective, yet adverse effects such as headaches and cognitive impairment often limit its tolerability (8, 9). Repetitive transcranial magnetic stimulation (rTMS), as a non-invasive neurostimulation technique, modulates the excitability of the left temporo-parietal cortex. Preliminary studies suggest it has potential value in improving AH in schizophrenia and is well-tolerated, offering a new treatment option for treatment-resistant patients (10).

This report describes the case of a patient with refractory auditory hallucinations who did not respond to multiple antipsychotic medications and experienced intracranial hypertension syndrome following MECT treatment. Following combined rTMS therapy, the patient’s AH improved significantly. The aim of this report is to provide new clinical insights for patients facing similar treatment challenges.

## 2 Case presentation

### 2.1 Chief complaints

The 21-year-old female patient experienced auditory hallucinations and persecutory delusions for more than 5 years.

### 2.2 History of present illness

Five years ago, without any apparent trigger, the patient gradually developed insomnia, persecutory delusions, referential delusions, and auditory hallucinations (AH), accompanied by emotional instability and irritability. During the first year of illness, she had systematically tried risperidone, ziprasidone, and sulpiride at adequate doses for at least 6 to 8 weeks each, but the results were poor. Two years prior to admission, quetiapine was also tried without satisfactory response. Approximately one year prior to admission, she transitioned to combination therapy with olanzapine and lurasidone; aripiprazole was added in the previous six months. Given her significant weight gain and dyslipidemia, clozapine had not been previously administered. After these medication trials, her mood stabilized somewhat and her feelings of persecution diminished; however, the AH persisted day and night without relief, severely impairing her social functioning and sleep quality, and rendering her unable to work normally. Failure to achieve adequate symptom remission despite multiple antipsychotic trials administered at therapeutic doses and for sufficient duration (at least 6–8 weeks each) fulfilled operational criteria for treatment-resistant schizophrenia. Notably, treatment resistance primarily reflected the persistence of auditory hallucinations, whereas persecutory delusions and mood symptoms showed partial improvement. Over the past year on medication, the patient gained approximately 10 kg and developed adverse reactions including limb rigidity, bradykinesia, and memory impairment. On admission, the medication regimen was as follows: aripiprazole 15 mg/day, olanzapine 15 mg/day, lurasidone 80 mg/day, and trihexyphenidyl 4 mg/day.

### 2.3 History of past illness

The patient had a history of migraine but no history of other medical conditions.

### 2.4 Personal and family history

The patient’s grandfather had a history of mental illness; other family members reported no history of migraine or mental illness.

### 2.5 Physical examination

Vital signs were stable upon admission. The patient had a body mass index (BMI) of 33.35 kg/m². A neurological examination revealed resting tremors in both hands, slowed movements, and slightly increased muscle tone.

### 2.6 Laboratory examinations

On admission, complete blood count, fasting blood glucose, and liver and kidney function tests were all normal. Abnormal lipid metabolism was observed.

### 2.7 Imaging examinations

Cranial magnetic resonance imaging (MRI) revealed no abnormal signals or space-occupying lesions, and both the electroencephalogram and electrocardiogram were normal. Funduscopic examination showed no papilledema in either eye.

## 3 Treatment

After admission, the patient underwent medication optimization from February 9 to 12. The doses of aripiprazole and lurasidone were reduced. While the body tremors improved, the auditory hallucinations (AH) remained unchanged. From February 13 to 22, to enhance the anti-hallucinatory effect, the dose of olanzapine was increased to 20 mg/day, lurasidone and aripiprazole were discontinued, and blonanserin was added (gradually titrated to 12 mg/day). During this period, the patient experienced sluggishness and constipation; due to poor efficacy of these medications, the patient underwent the first session of MECT on February 23. MECT was performed using bilateral frontotemporal electrode placement. Anesthesia was induced with propofol 1.5 mg/kg, and succinylcholine 1.0 mg/kg was administered for muscle relaxation. A generalized tonic-clonic seizure lasting approximately 38 seconds was observed clinically. Peri-procedural monitoring revealed stable vital signs, with blood pressure and heart rate remaining within normal ranges throughout the procedure. Following the procedure, the patient experienced severe headache (Numerical Rating Scale (NRS) 7), accompanied by two episodes of projectile vomiting and diplopia. A weak positive meningeal irritation sign was noted. Following consultation with the neurology department, intracranial hypertension syndrome was suspected. The headache resolved completely by the following day; cranial MRI showed no abnormalities, and fundoscopic examination revealed no papilledema in both eyes. As the patient could not tolerate MECT, the treatment was discontinued. Following 2 weeks of ineffective treatment with olanzapine combined with blonanserin, rTMS was initiated on March 2. A standard figure-8 coil was used. The coil was positioned using the international 10–20 EEG system, with the stimulation target centered at the midpoint between T3 and P3 over the left temporo-parietal cortex. Low-frequency (1 Hz) stimulation was applied once daily, with 1,500 pulses per session lasting 25 minutes. Seven sessions were administered over 7 consecutive days. The initial stimulation intensity was set at 80% of the motor threshold. Mild headache (NRS 3) occurred after the first session; the headache resolved after adjusting the intensity to 74%. AH decreased significantly after 3 days of treatment; after 1 week of treatment, the AH completely resolved, and the patient’s mood stabilized.

## 4 Diagnosis

Based on the patient’s medical history, clinical presentation, treatment course, and response to treatment, the final diagnosis was: 1. Treatment-resistant schizophrenia (with refractory auditory hallucinations); 2. Extrapyramidal reactions; 3. Presumed acute intracranial hypertension syndrome; 4. History of migraine.

## 5 Outcome and follow-up

After 1 week of continuous rTMS treatment, the auditory hallucinations (AH) completely disappeared, the patient’s mood stabilized, and social functioning gradually improved. Prolactin levels remained elevated, likely attributable to ongoing antipsychotic medication. There was no lactation; the patient was monitored. At the follow-up visit 3 weeks later, the patient’s condition remained stable, and the AH had not recurred. The patient reported that the disappearance of hallucinations markedly improved her sleep and daily functioning, and she expressed satisfaction with the rTMS treatment.

## 6 Discussion

This report describes a successful transition from MECT to rTMS in a 21-year-old female patient with a 5-year history of schizophrenia characterized by auditory hallucinations (AH). Despite treatment with multiple antipsychotic agents, her hallucinations remained refractory. MECT was discontinued due to intracranial hypertension syndrome. Following rTMS treatment, the patient achieved complete resolution of AH, along with mood stabilization and gradual improvement in social functioning.

Hallucinations are one of the core symptoms of schizophrenia, affecting more than half of patients (11, 12). Persistent AH is significantly associated with suicidal ideation and behavior (13), and due to their chronic and intrusive nature, they often lead to anxiety, depression, and physical discomfort (14). This patient’s illness spanned 5 years, and AH persisted despite combination therapy with multiple antipsychotic medications, meeting the diagnostic criteria for treatment-resistant schizophrenia (TRS) (15). According to treatment guidelines, clozapine is the first-line recommended medication for patients with TRS (16). However, 40%–70% of TRS patients show poor response to clozapine or are unable to tolerate its adverse effects (17, 18). The patient in this case had already experienced weight gain and dyslipidemia, with a BMI of 33.35 kg/m², indicating a high risk of metabolic syndrome; therefore, clozapine was not selected in the clinical decision-making process. Although studies suggest prioritizing medications with fewer metabolic side effects, such as lurasidone and aripiprazole, for patients at risk of metabolic syndrome (19), these agents had not yielded satisfactory therapeutic outcomes during the patient’s prior treatment; consequently, further exploration of alternative treatment options was warranted.

A significant physiological event during MECT is transient intracranial hypertension, a response that may be more pronounced in patients with hypertension (20). Although this physiological increase in intracranial pressure is well recognized and documented, to our knowledge, no reports have described MECT-induced intracranial hypertension accompanied by overt clinical symptoms; the mechanism underlying this symptomatic presentation remains unclear. One prospective study suggests that electroconvulsive therapy (ECT) itself does not appear to elevate intracranial pressure, but that increases may occur following anesthetic induction and muscle relaxation (21). More recent research indicates that ECT may increase cerebral blood flow and oxygen consumption, which in turn could raise intracranial pressure (22). This physiological change is well tolerated in most patients; however, it may precipitate serious complications in individuals with preexisting intracranial conditions, such as space−occupying lesions, cerebrovascular disease, or hydrocephalus, in whom baseline intracranial pressure is already elevated or compensatory mechanisms are impaired (23). Additionally, a study by Spoor et al. demonstrated that intracranial pressure rises transiently during seizure induction by ECT (24).

The patient had no history of hypertension, ruling out elevated blood pressure as a cause of increased intracranial pressure. While cranial MRI revealed no structural abnormalities, the possibility of occult vascular lesions, such as microvascular malformations or arterial stenosis undetectable by conventional MRI, cannot be completely excluded. Alternatively, dysregulation of cerebrospinal fluid dynamics and alterations in venous sinus pressure may also contribute to elevated intracranial pressure (25). These pathophysiological changes frequently present with unremarkable neuroimaging and normal cerebrospinal fluid profiles (26), contributing to the misdiagnosis of approximately two-thirds of affected cases as migraine or probable migraine (27). Lumbar puncture was not performed, as performing it under sedation was considered unsafe given the suspected acute intracranial hypertension with risk of herniation, and the potential for sedation to mask subsequent neurological assessment; however, the patient exhibited typical manifestations of intracranial hypertension following MECT, consistent with the recognized clinical manifestations of acute intracranial hypertension (28), supporting a presumptive diagnosis. Although the patient had a history of migraine, her typical episodes had never involved projectile vomiting, diplopia, or meningeal signs. The acute onset of these neurological symptoms immediately following MECT, combined with the exclusion of structural lesions by MRI and chronic intracranial hypertension by fundoscopy, rendered a migraine attack unlikely and supports the clinical diagnosis of acute intracranial hypertension syndrome. According to the modified Dandy criteria for intracranial hypertension, the patient fulfilled criteria related to symptoms and signs of elevated intracranial pressure (including severe headache, projectile vomiting, diplopia, and meningeal signs), as well as the absence of structural abnormalities on neuroimaging. However, elevated cerebrospinal fluid opening pressure could not be confirmed because lumbar puncture was not performed. Therefore, the diagnosis should be regarded as presumptive rather than definitive. Given this, we recommend maintaining a high level of vigilance for post-MECT headache; when headache progressively worsens or is accompanied by vomiting or visual disturbances, prompt repeat fundoscopic examination should be performed. If papilledema is absent, lumbar puncture may then be considered.

As a non-invasive neurostimulation technique, rTMS modulates cortical excitability in specific brain regions; its efficacy in treating negative symptoms of schizophrenia has been widely demonstrated (29), but its effectiveness in treating AH remains controversial. Research by Slotema et al. suggests that low-frequency (1 Hz) rTMS may not be an effective treatment strategy (30), and a systematic review indicates that current evidence is insufficient to support the notion that rTMS provides additional benefits when used as an adjunct to antipsychotic medication (31). Nevertheless, further studies have shown that image-guided rTMS can safely and effectively alleviate AH in patients with schizophrenia, including those resistant to clozapine (10, 32). The strongest current evidence supports the use of 1 Hz rTMS stimulation of the left temporo-parietal cortex for the short-term treatment of persistent AH (33). In this case, the patient received 1 Hz low-frequency stimulation to the left temporo-parietal cortex. Headaches occurred after the first treatment session; this resolved after reducing the stimulation intensity from 80% to 74% of the motor threshold, and the patient tolerated subsequent treatments well. On day 3 of treatment, the patient reported a marked reduction in auditory hallucinations, which completely resolved after 1 week of treatment. This rapid and significant therapeutic effect further supports the clinical value of rTMS in the treatment of refractory auditory hallucinations. It is worth noting that the patient continued a medication regimen of olanzapine combined with blonanserin throughout the rTMS treatment period. The improvement in AH may be partly attributed to the ongoing effects of the medication; however, considering that the previous course of 2 weeks of medication failed to alleviate the hallucinations, whereas they rapidly resolved after initiating rTMS treatment, these observations suggest that rTMS played a key role in this treatment response.

This case report has the following limitations: The diagnosis of acute intracranial hypertension syndrome remains presumptive, as confirmatory lumbar puncture with opening pressure measurement was not performed; the follow-up period was limited to 3 weeks, and no maintenance rTMS sessions were planned at discharge, precluding assessment of the long-term durability of the rTMS response. Long-term observation is warranted in future cases. The absence of standardized measures of auditory hallucinations by validated instruments limits the objectivity of treatment-response assessment and reduces comparability with previous studies. Future case reports and clinical investigations should incorporate standardized symptom measures whenever feasible.

## Data Availability

The original contributions presented in the study are included in the article/supplementary material. Further inquiries can be directed to the corresponding author.
